# Supplementary material for: De novo variants in the splicing factor gene SF3B1 are associated with neurodevelopmental disorders
Source: Nat Commun. 2026 Jan 23;17:1569. doi: 10.1038/s41467-026-68284-9 (PMC12902031; doi:10.1038/s41467-026-68284-9)
Supplement: Supplementary file 1 — Supplementary Information [file 41467_2026_68284_MOESM1_ESM.pdf]

Supplementary information

"**De novo variants in the splicing factor gene *SF3B1* are associated with neurodevelopmental disorders**", by K. Uguen, T. Bergot *et al.*

Supplementary Figures

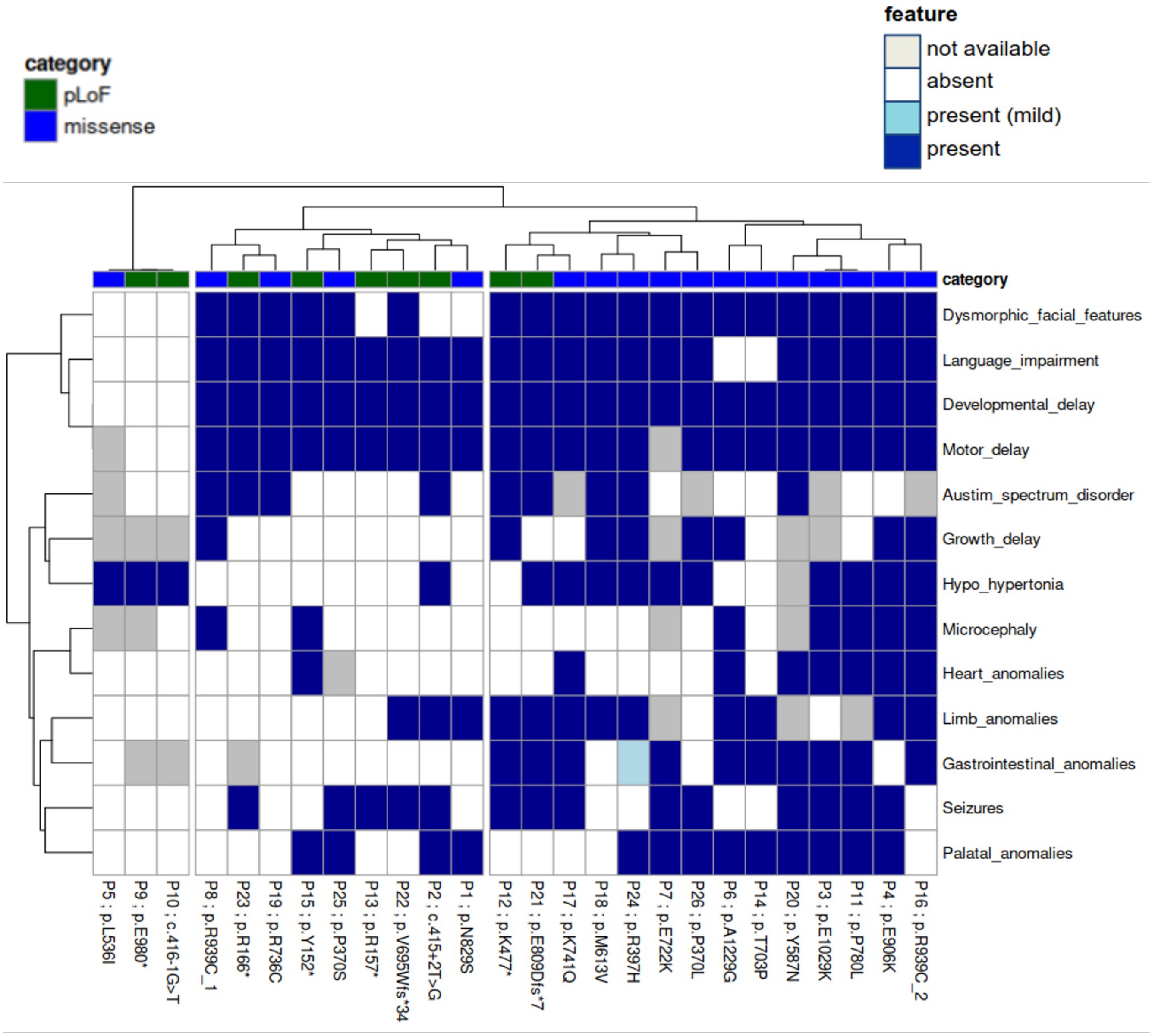

Supplementary Figure 1. Hierarchical clustering of clinical features (rows) of the cohort's patients (columns). Features with missing data in >30% of patients were excluded.

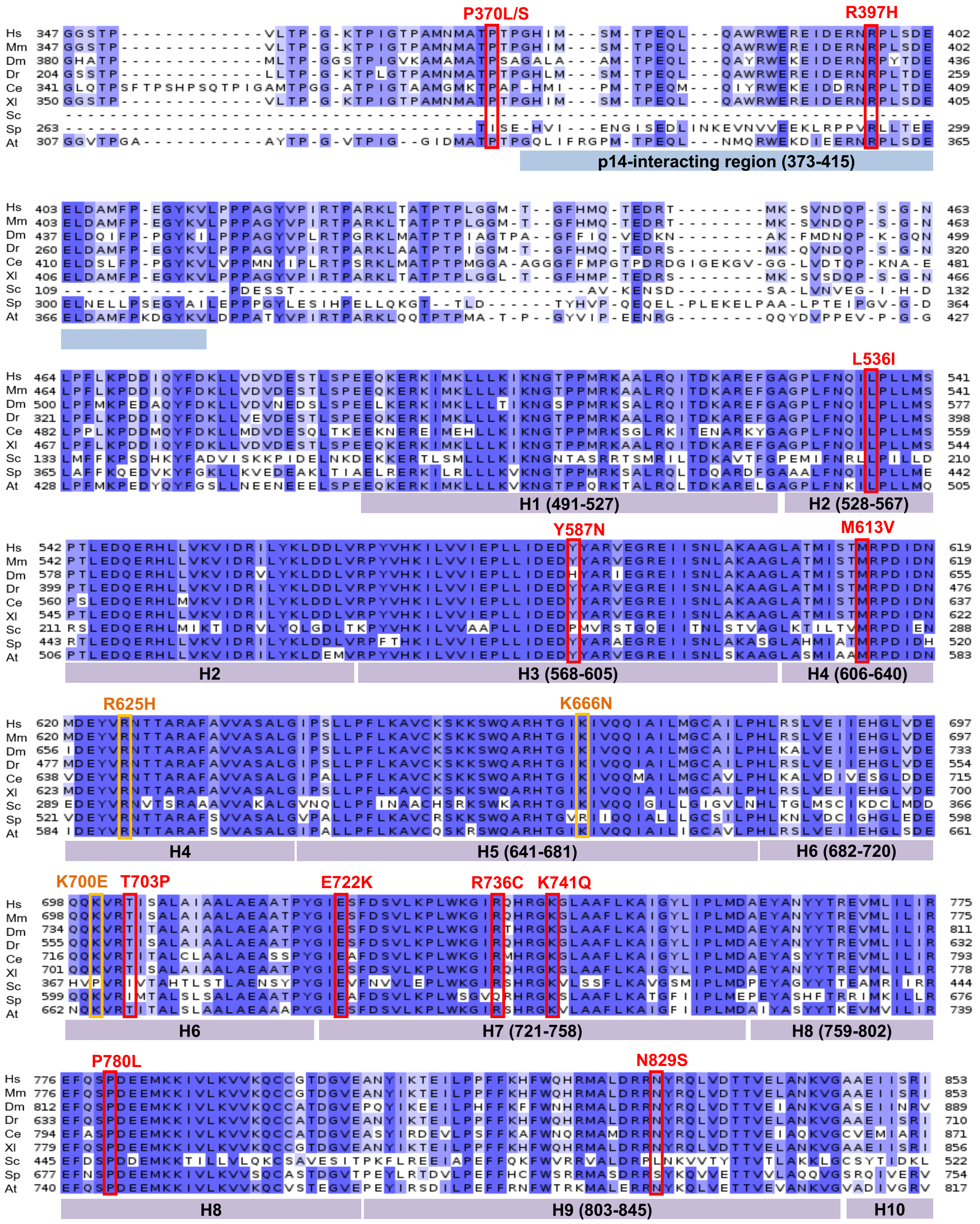
P370L/S
R397H
p14-interacting region (373-415)
L536I
H1 (491-527)
H2 (528-567)
Y587N
M613V
H2
H3 (568-605)
H4 (606-640)
R625H
K666N
H4
H5 (641-681)
H6 (682-720)
K700E
T703P
E722K
R736C
K741Q
H6
H7 (721-758)
H8 (759-802)
P780L
N829S
H8
H9 (803-845)
H10
Hs
Mm
Dm
Dr
Ce
Xl
Sc
Sp
At

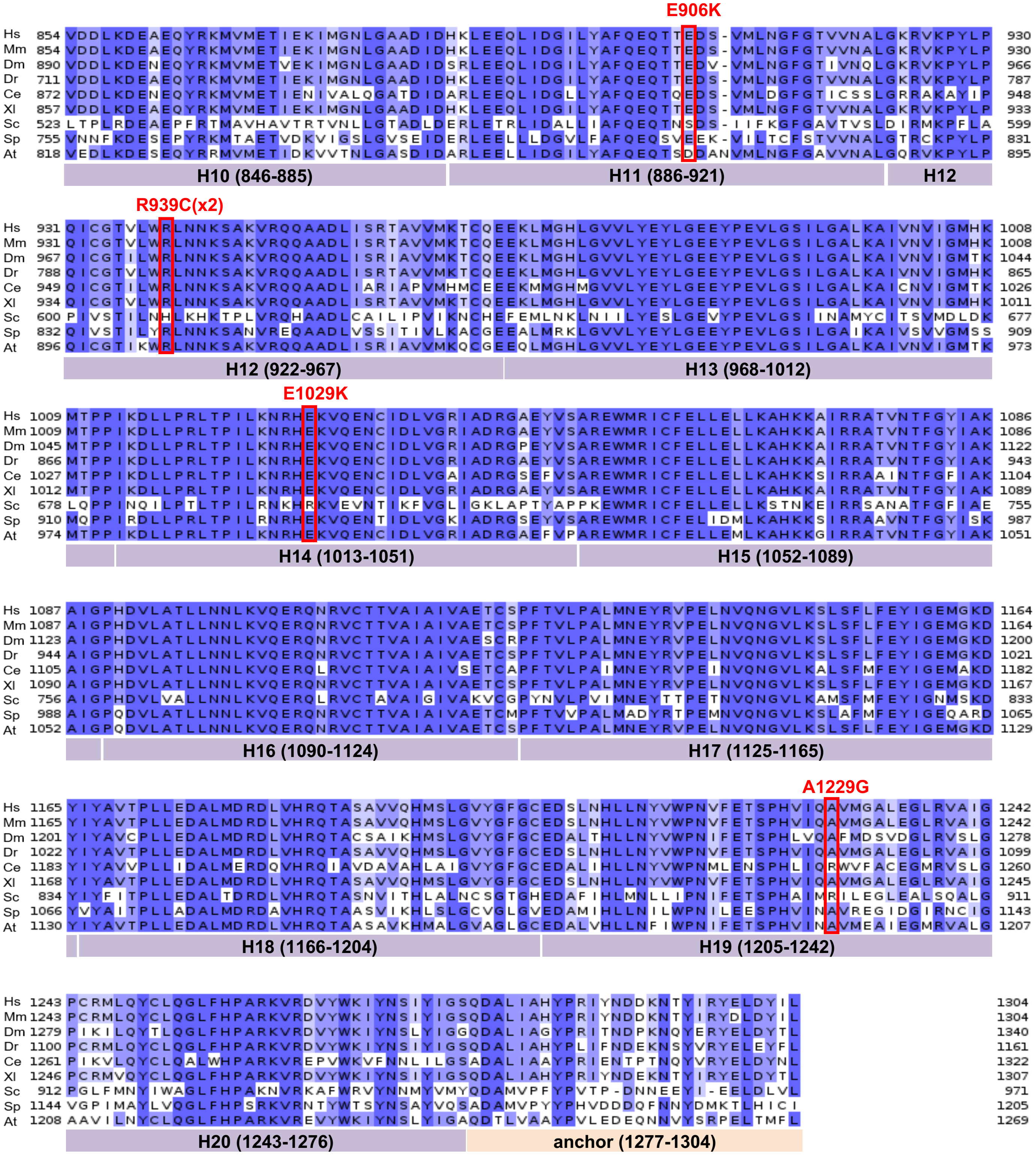

Supplementary Figure 2. Multiple sequence alignment of SF3B1 proteins from different species. Missense variants from the cohort and the cancer-associated variants K700E, R625H and K666 are represented in red and orange, respectively. H1-H20 - HEAT repeats 1-20 of SF3B1 according to Cretu et al[4]. *Hs - Homo sapiens; Mm - Mus musculus; Dm - Drosophila melanogaster; Dr - Danio rerio; Ce - Caenorhabditis elegans; Xl - Xenopus laevis; Sp - Schizosaccharomyces pombe; Sc - Saccharomyces cerevisiae; At - Arabidopsis thaliana.*

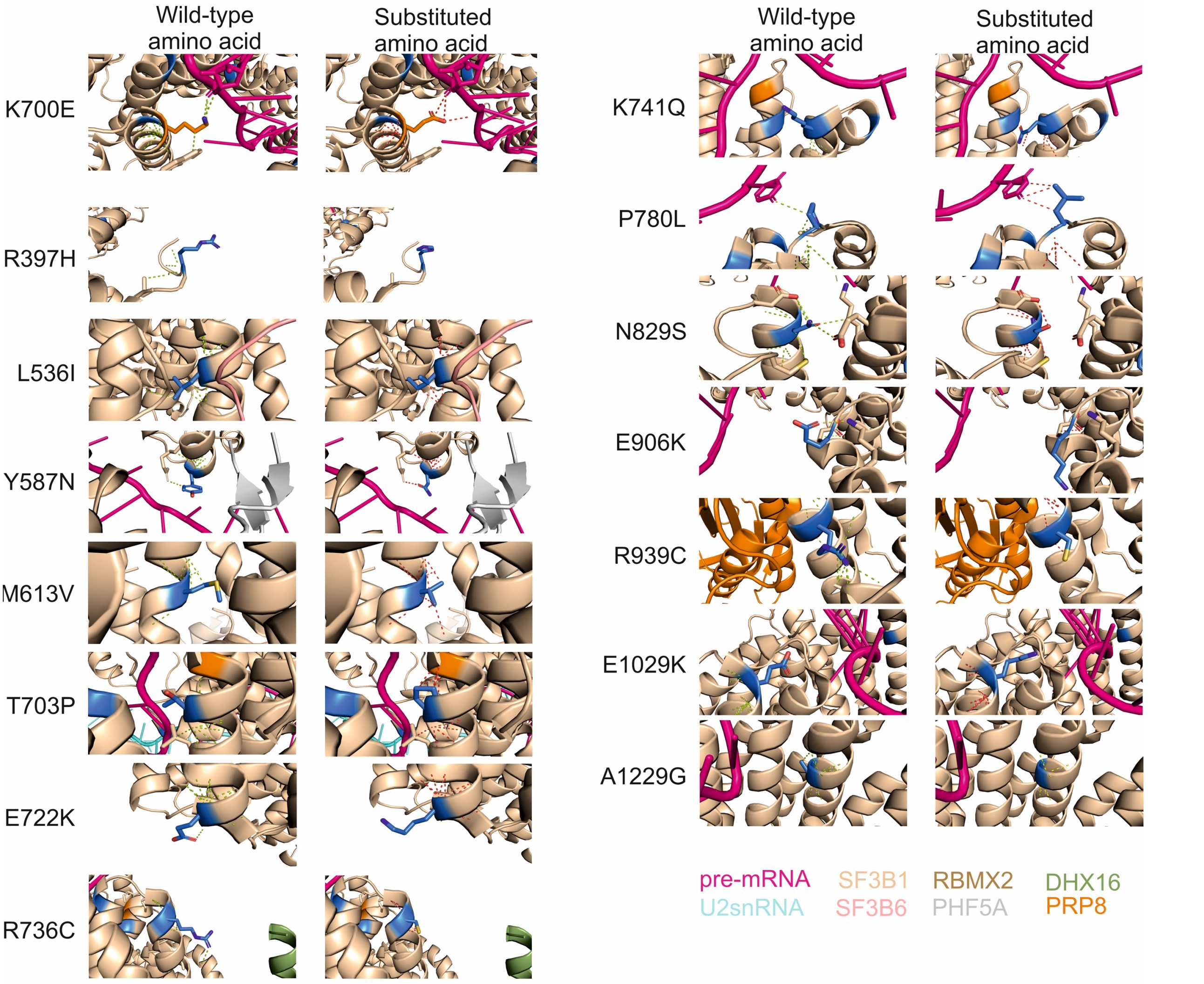

Supplementary Figure 3. Spatial vicinity of the residues that are substituted, some of which may be involved in intermolecular contacts. The NDD-associated variants are indicated in blue and the somatic variant K700 in orange. Distances below 3.5 A between amino acids of interest and neighbouring atoms are represented as dashes in green for wild-type and red for the substituted amino acid. Colour legend: SF3B1 (beige), SF3B3 (black), SF3B6 (pink), RBMX2 (light brown), DHX16 (green), PRP8 (orange), pre-mRNA (pink), U2 (cyan).

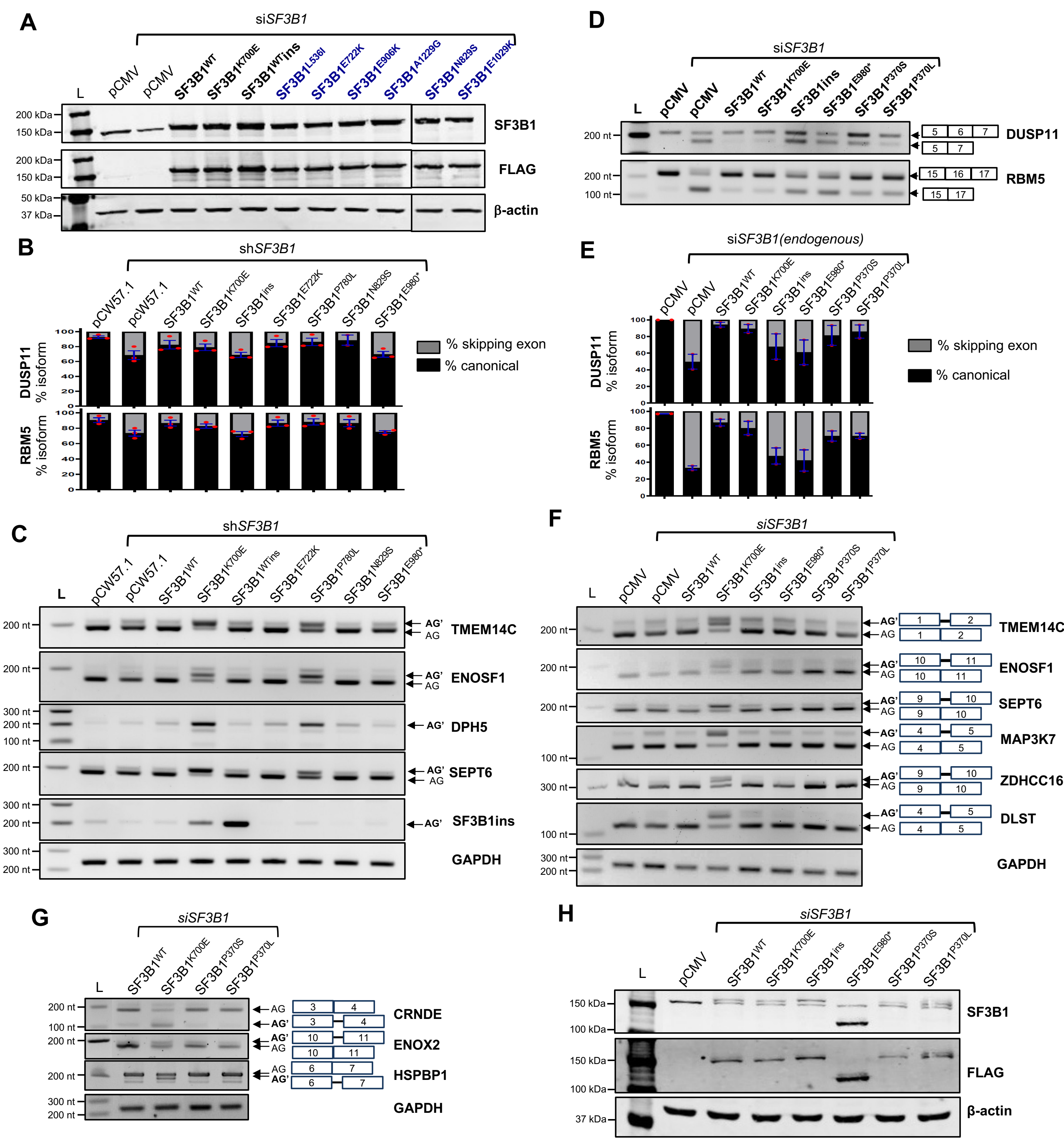

Supplementary Figure 4. Additional targeted analysis of splicing events driven by *SF3B1* missense variants, including P370S and P370L, in K562 cells. A. Steady-state levels of SF3B1 protein detected by Western-Blot upon transient expression of distinct *SF3B1* variants in K562 cells. Total SF3B1 proteins (endogenous and recombinant) were detected using anti-SF3B1 antibody. Recombinant SF3B1 was detected using anti-FLAG antibody. **B**. Digital quantification of exon skipping events in DUSP11 and RBM5, in K562 expressing *SF3B1* variants of interest under the control of a dox-inducible promoter, together with endogenous *SF3B1* silencing (n=3). **C.** RT-PCR detection of aberrant transcripts known to be specifically produced upon expression of cancer-associated *SF3B1* mutations (K700E) in the same cells. **D.** Analysis of exon skipping of *DUSP11* and *RBM5* in K562 cells co-expressing si*SF3B1* and P370S and P370L variants (48h post-transfection). **E.** Digital quantification of *DUSP11* and *RBM5* exon skipping (n=2). **F.** RT-PCR detection of aberrant transcripts known to be specifically produced upon expression of cancer-associated *SF3B1* mutations (K700E). **G.** Effect of P370S and P370L variants on the splicing of *CRNDE*, *ENOX2* and *HSPBP1*. **H.** Steady-state levels of SF3B1 protein detected by Western-Blot upon transient expression of distinct *SF3B1* variants in K562 cells (as in A).

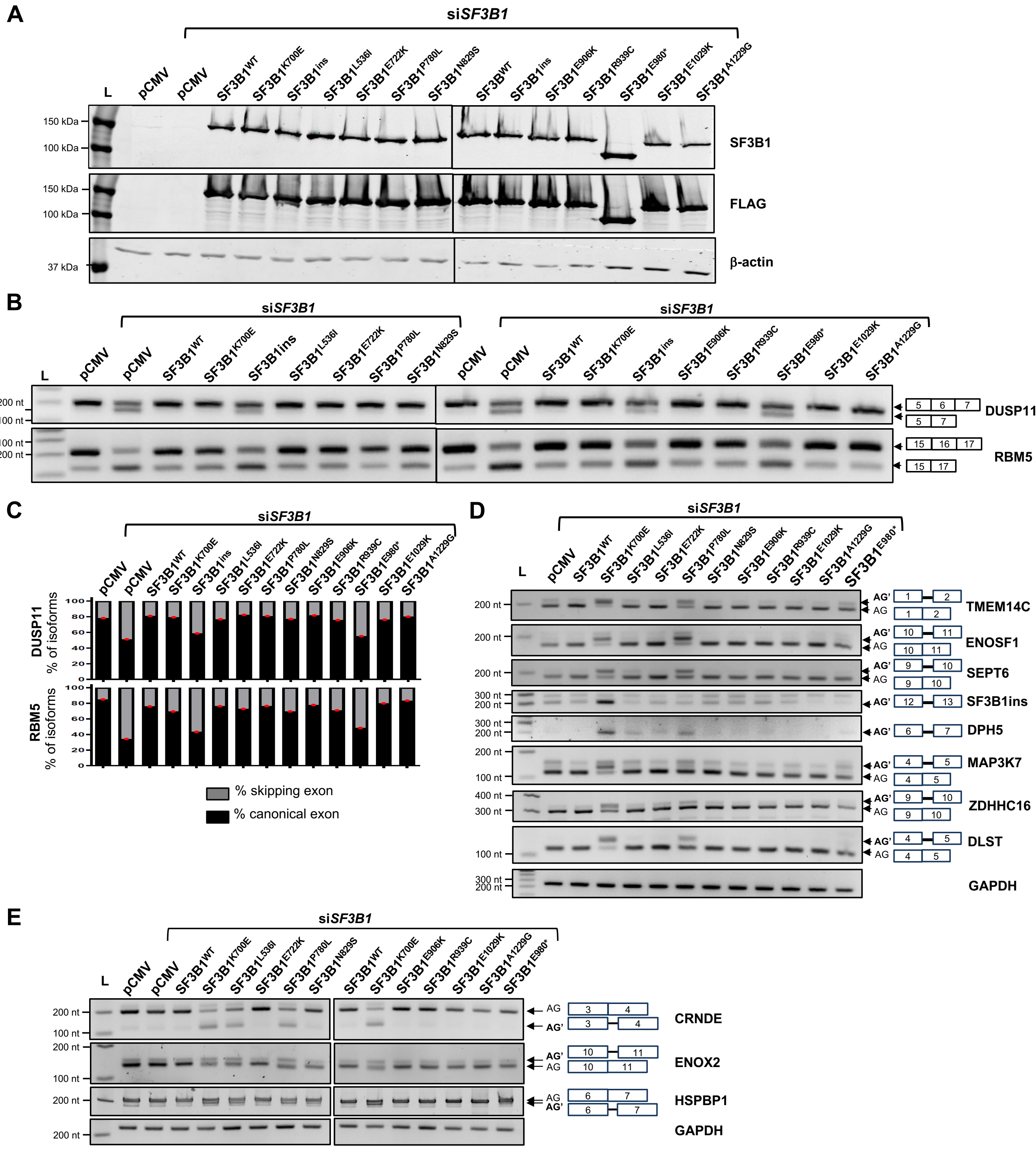

Supplementary Figure 5. Molecular impact of *SF3B1* missense variants in HEK293T cells.
A. Steady-state levels of SF3B1 protein detected by Western-Blot upon transient expression of distinct *SF3B1* variants in HEK293T cells. Total SF3B1 proteins (endogenous and exogenous) were detected using anti-SF3B1 antibody. Plasmid-encoded *SF3B1* was detected using anti-FLAG antibody. **B.** Analysis of exon skipping of *DUSP11* and *RBM5*, used as markers of *SF3B1* loss of function, in HEK293T cells co-expressing si*SF3B1* and various NDD-associated variants (48h post-transfection). **C.** Digital quantification of *DUSP11* and *RBM5* exon skipping. **D.** RT-PCR detection of aberrant transcripts known to be specifically produced upon expression of cancer-associated *SF3B1* mutations (K700E), in HEK293T transfected cells. **E.** Effect of NDD-associated variants on the splicing of *CRNDE*, *ENOX2* and *HSPBP1* in HEK293T cells (at 48h post-transfection).

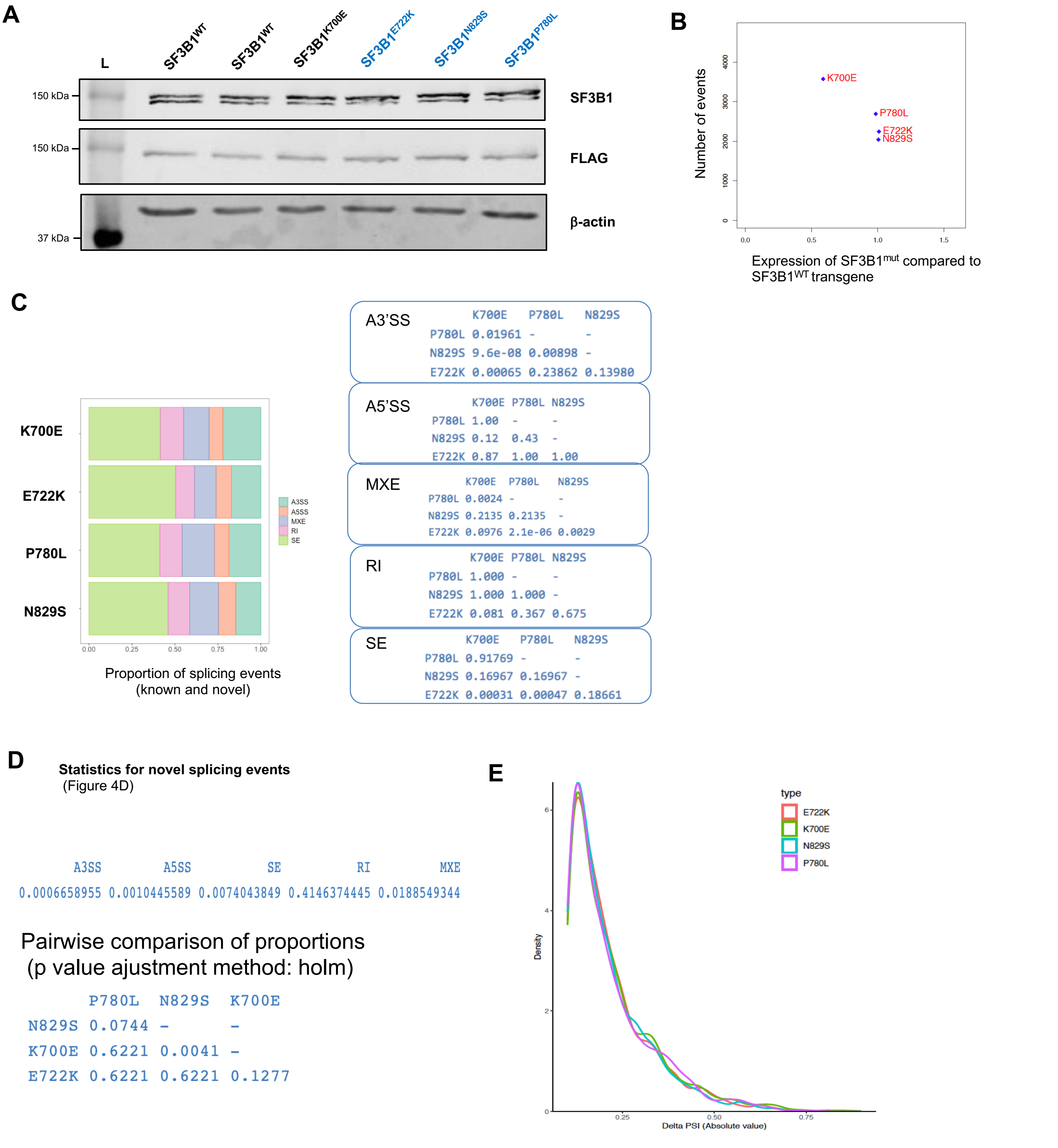

Supplementary Figure 6. Supplemental controls of RNAseq experiments.
A. Representative western-blot showing equivalent levels of recombinant SF3B1 in the K562 cell samples (inducible expression of SF3B1 variant for 7 days) used for RNA sequencing. **B.** Number of splicing events in function of transgene transcript levels. P780L, E722K and N829S are expressed at a level similar to the wild type transgene (ratio = 1). **C.** Proportion of known and novel splicing events, with pairwise comparison of proportions (p value adjustment method). **D.** Statistics for novel splicing events only (cf Figure 4D). **E**. Graph representing the density of reads in function of the absolute value of delta PSI (PSI SF3B1var - PSI SF3B1wt) for all splicing events.

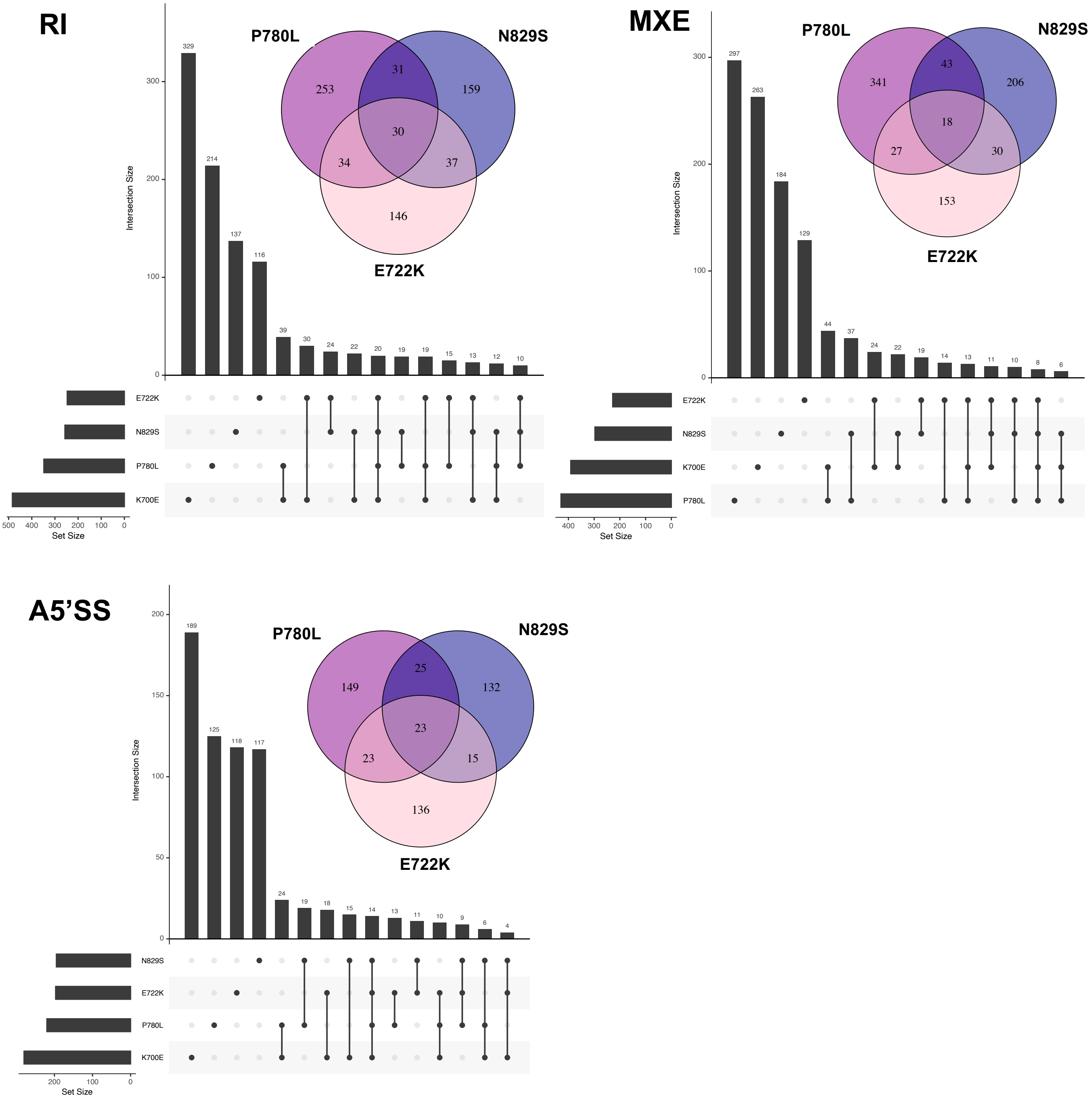

Supplementary Figure 7. Analysis of RI, MXE and A5’SS splicing events Venn diagram and Upset Plots visualising the number of differentially spliced junctions that are in common between NDD-associated *SF3B1* variants (Venn) and K700E (Upset Plots) for RI, MXE and A5’SS events.

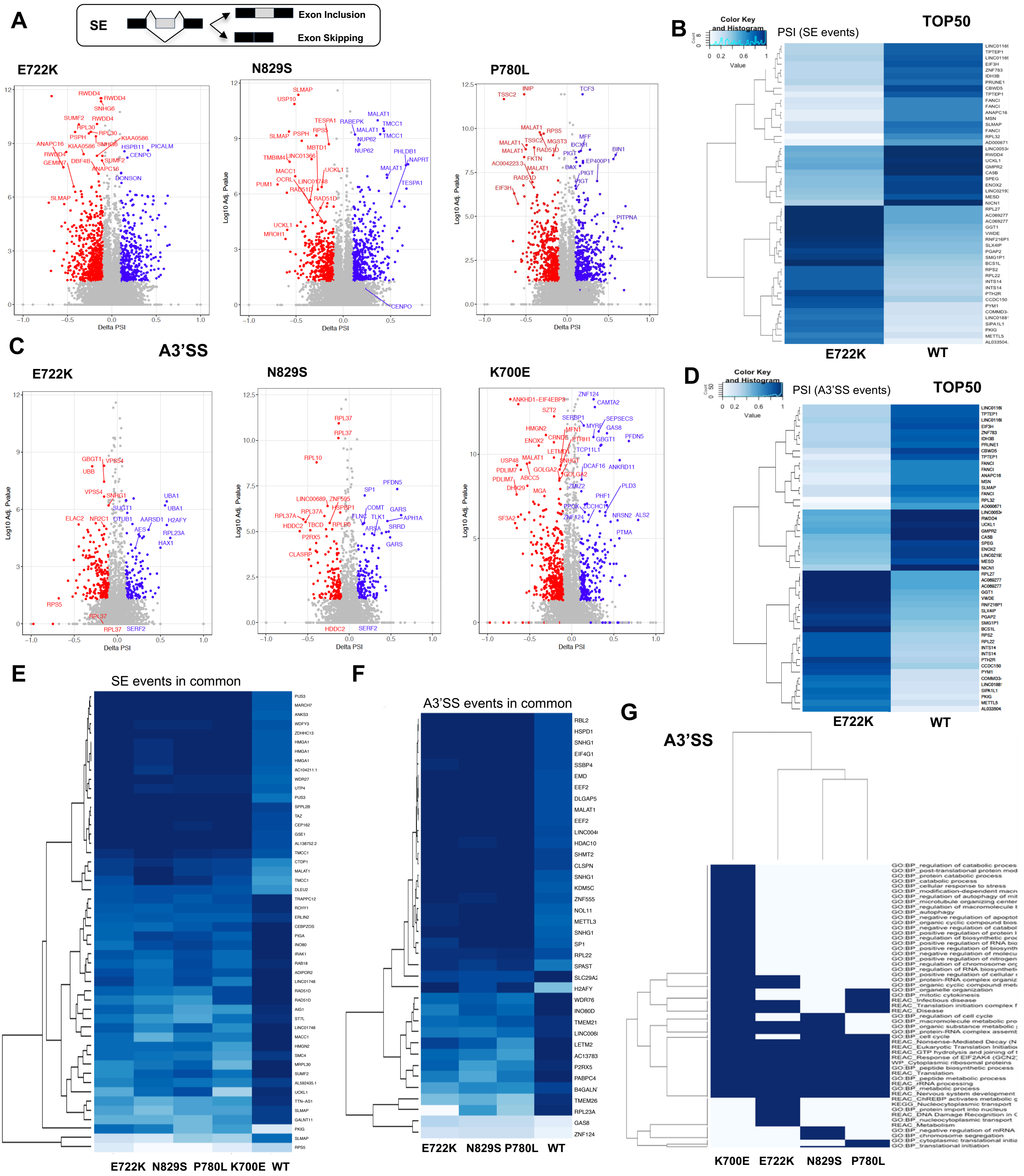

Supplementary Figure 8. Supplemental analysis of Skipping Exons (SE) and Alternative 3' Splice Sites (A3'SS) events resulting from the transcriptomic analysis. **A.** Volcano plots showing the significant differential SE events upon expression of E722K, N829S and P780L variants in comparison to the wild type counterpart. **B.** Heat map representing the top50 differential SE events in E722K compared to wild type. The color intensity reflects the value of Percent Spliced In (PSI) index. **C.** Volcano plots showing the significant differential A3'SS events upon expression of E722K, N829S and K700E variants in comparison to the wild type counterpart. **D.** Heat map representing the top50 A3'SS events in E722K compared to wild type. The color intensity reflects the value of Percent Spliced In (PSI) index. **E.** Heat map showing the PSI of differential Skipping Exons events in common between NDD-associated variants and K700E. **F.** Heat map showing the Percent Spliced In index (PSI) of differential A3'SS events in common between NDD-associated variants. **G.** Heatmap resulting from the functional enrichment analysis applied on A3'SS events (with FDR<0.05).

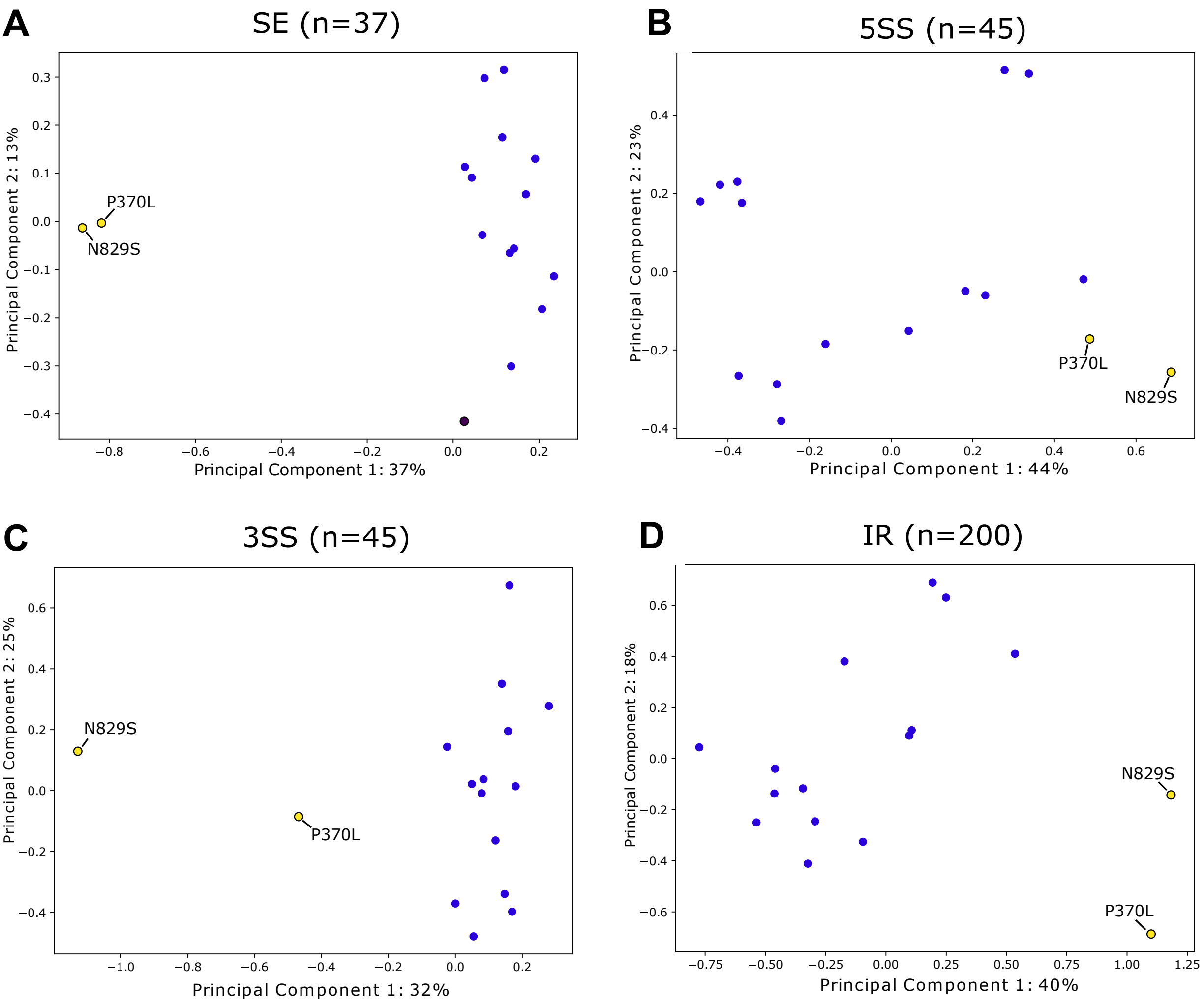

Supplementary Figure 9. Principal component analysis performed on PSI values of significant alternative splicing events from short-term cultured lymphocytes. RNA-Seq were performed on P1 and P26, harboring the variants p.N829S and p.P370L, respectively. They were compared with rmats-turbo to 14 controls (NDD without any SF3B1 variant) sequenced in the same run. PCA were performed using the PSI values of significant events for (A) alternative exon skipping (n=37), (B) alternative 5' splice sites (n=45), (C) alternative 3' splice sites (n=45), (D) intronic retentions (n=200). Blue: controls, yellow: SF3B1 individuals.

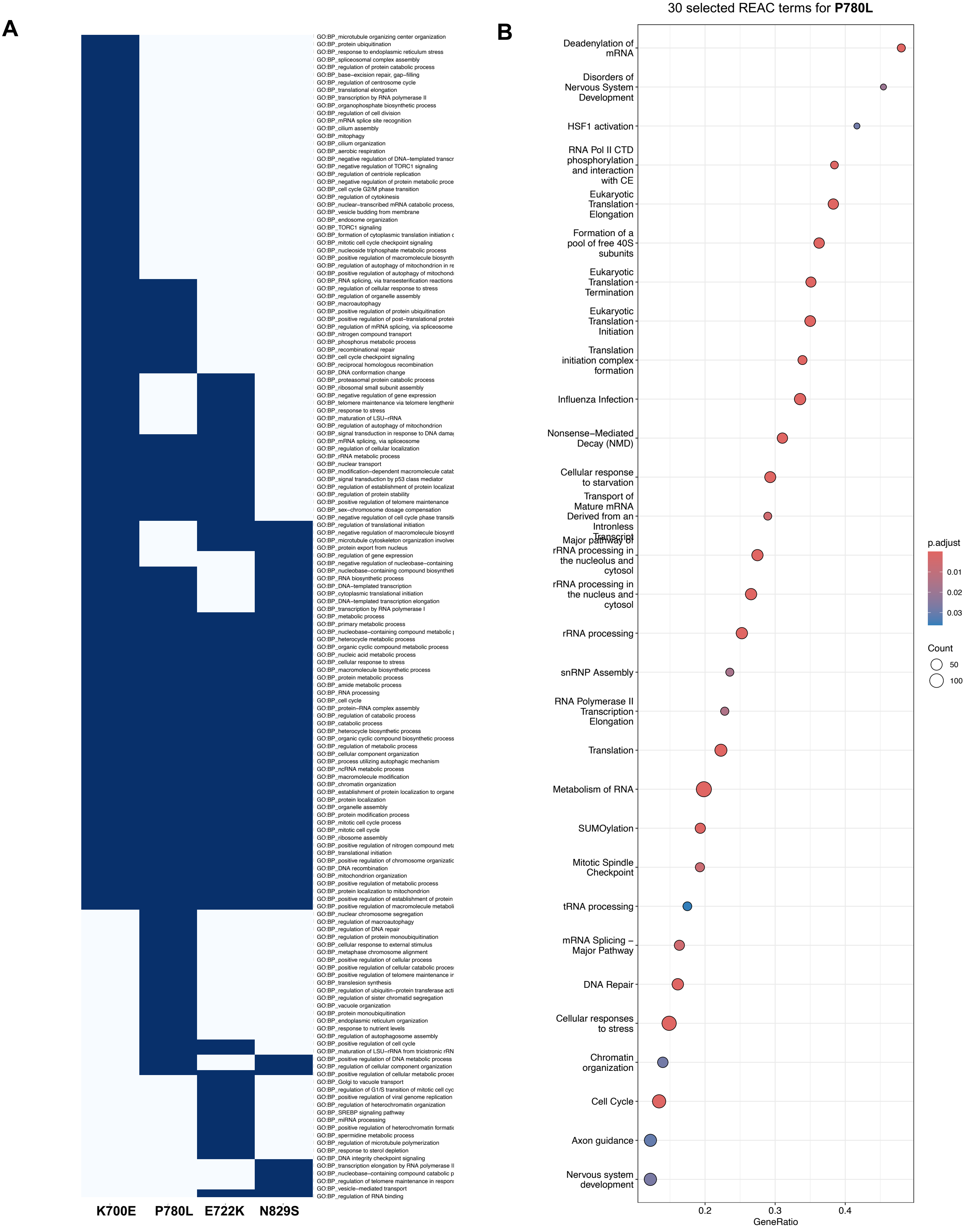

Supplementary Figure 10. GSEA analysis on differentially spliced genes. A. Heatmap resulting from the functional enrichment analysis applied on all differential splicing events for GO terms (with FDR<0.05). Only terms that contain less than 600 items were included in order to be more specific. **B.** Dot-plot highlighting 30 selected enriched REAC terms for the differential splicing events reported in the P780L condition.

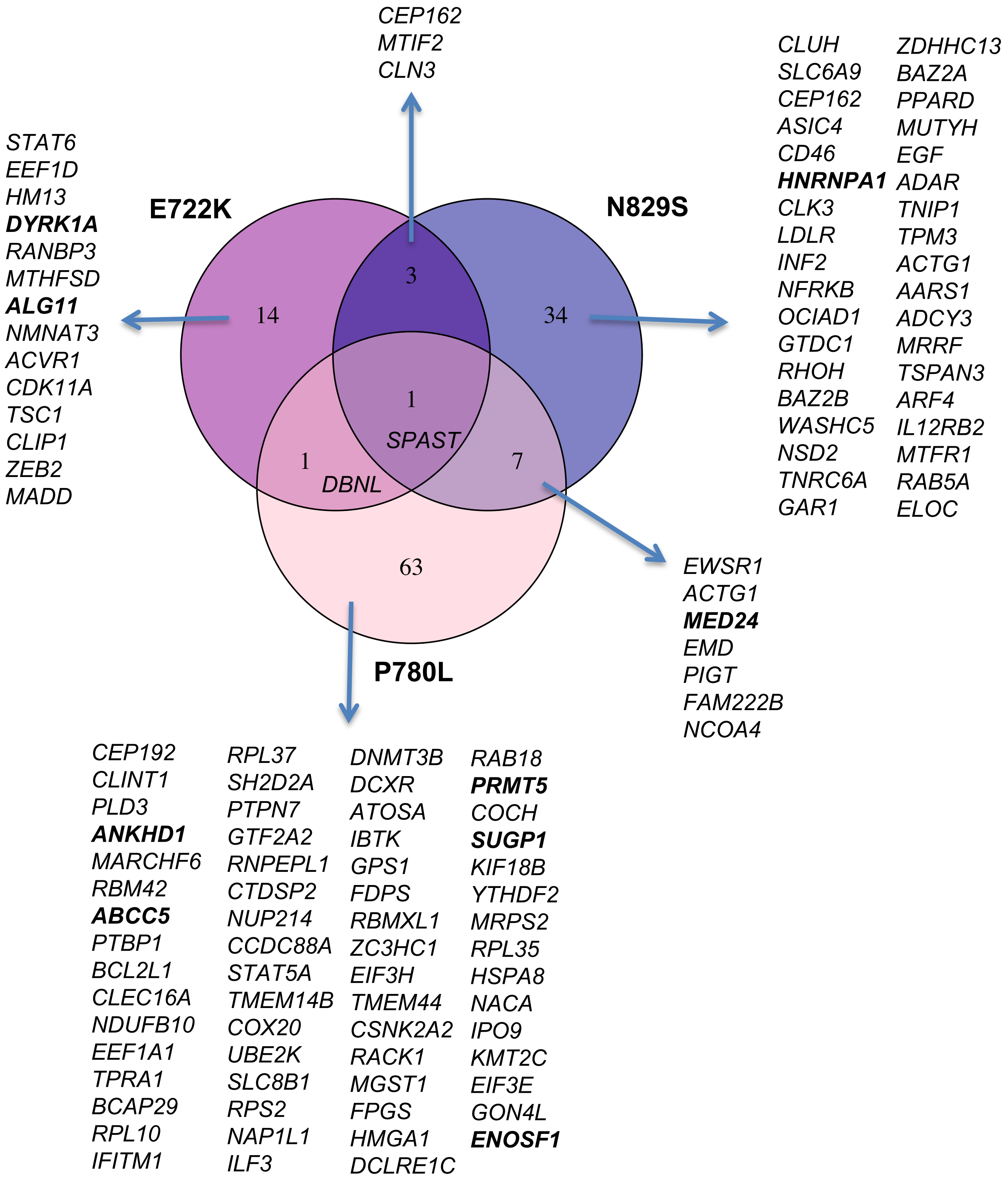

Supplementary Figure 11. Venn diagram visualising the number of splicing events overlapping with differentially expressed transcripts that are in common between NDD-associated *SF3B1* variants.

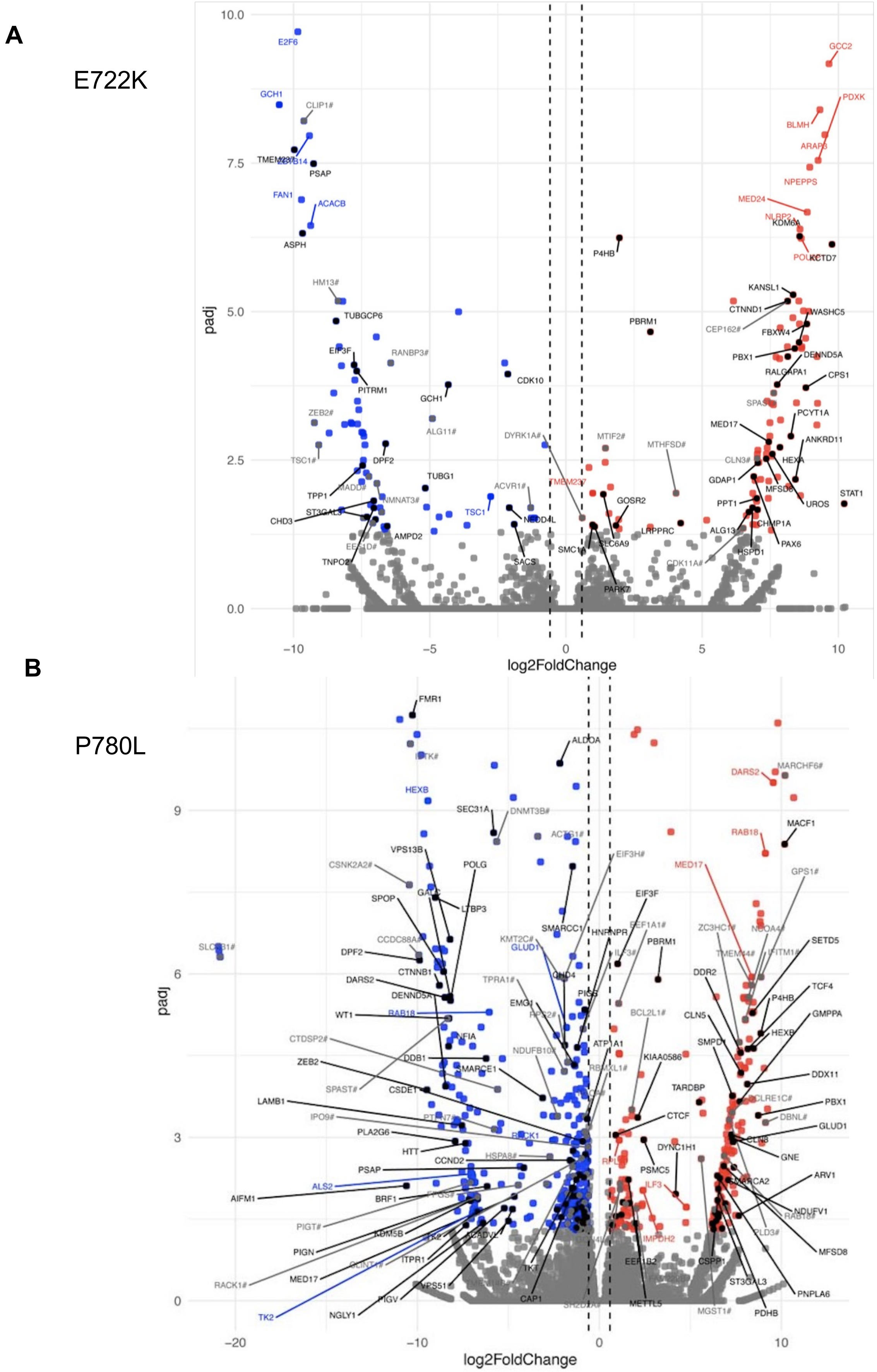

Supplementary Figure 12. Differentially expressed (DE) transcripts upon expression of E722K and P780L variants. Volcano plots showing the significant DE transcripts upon expression of E722K (**A**) or P780L (**B**), in comparison to the wild type counterpart. The # sign indicates DEG that also presents a differentially spliced event. The black colour indicates genes known to be associated with neurodevelopmental disorders.

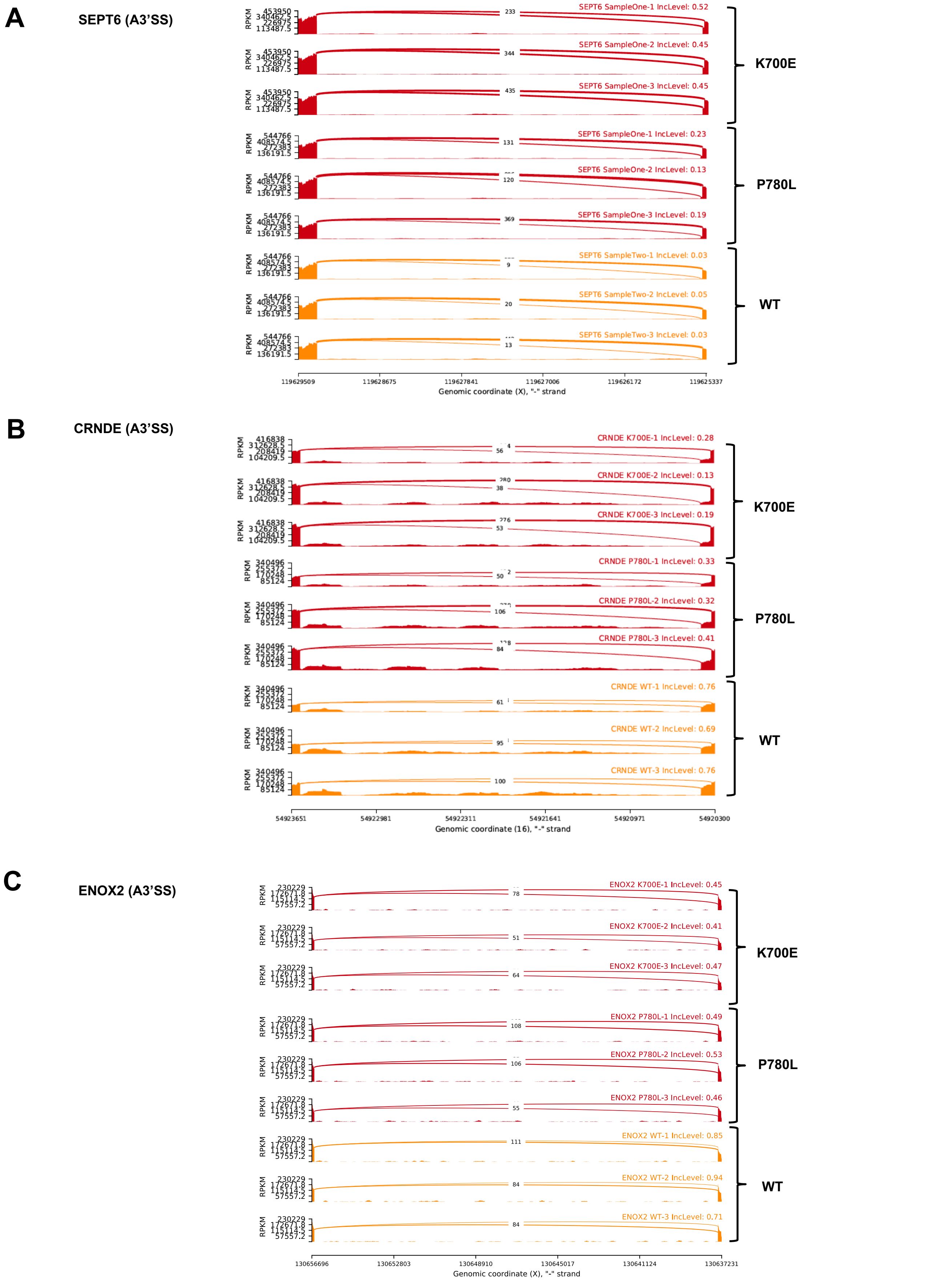

Supplementary Figure 13. Sashimi plots representing read coverage in RPKM for A3'SS events in SEPT6 (**A**), CRNDE (**B**) and ENOX2 (**C**) in K562 cells with inducible expression of *SF3B1* variants (WT, P780L and K700E).

Supplementary Tables

Supplementary Table 1. *In silico* prediction of the splicing effect of *SF3B1* missense variants using the MobiDetails[1] platform to query the following algorithms: MaxEntScan (MES), SPiP, dbscSNV and spliceAI. SS = Splice Site, ADA = AdaBoost, RF = Random Forest, AG = Acceptor Gain, AL = Acceptor Loss, DG = Donor Gain, DL = Donor Loss.

Variant c. (NM_012433.3)	Variant p.	MES		SPiP		dbscSNV		spliceAI			
		5'SS	3'SS	score	interpretation	ADA	RF	AG	AL	DG	DL
NM_012433.3:c.2486A>G	p.N829S	0	0	08.04 % [04.67 % - 12.73 %]	No effect on splicing	0	0	0	0	0	0
NM_012433.3:c.3085G>A	p.E1029K	0	0	30.67 % [23.41 % - 38.71 %]	Alteration of an exonic splicing regulatory element	0	0	0	0	0	0
NM_012433.3:c.2716G>A	p.E906K	0	0	09.3 % [02.59 % - 22.14 %]	No effect on splicing	0,97	0,81	0	0	0	0
NM_012433.3:c.1606C>A	p.L536I	0	0	07.8 % [04.53 % - 12.37 %]	No effect on splicing	0	0	0	0	0	0
NM_012433.3:c.3686C>G	p.A1229G	0	0	05.1 % [02.47 % - 09.18 %]	No effect on splicing	0	0	0	0	0	0
NM_012433.3:c.2164G>A	p.E722K	-44% (WT 3.10, MUT 1.71)	0	05.1 % [02.47 % - 09.18 %]	No effect on splicing	0	0	0	0	0	0
NM_012433.3:c.2815C>T	p.R939C	0	0	03.43 % [01.39 % - 06.94 %]	No effect on splicing	0	0	0,01	0	0	0
NM_012433.3:c.2339C>T	p.P780L	0	0	07.62 % [04.42 % - 12.08 %]	No effect on splicing	0	0	0	0	0	0
NM_012433.3:c.2107A>C	p.T703P	0	0	03.59 % [01.56 % - 06.95 %]	No effect on splicing	0	0	0	0	0	0
NM_012433.3:c.2221A>C	p.K741Q	0	0	09.3 % [02.59 % - 22.14 %]	No effect on splicing	0,62	0,67	0	0	0	0
NM_012433.3:c.1837A>G	p.M613V	+209.21 (WT -3.91, MUT 4.27)	0	03.59 % [01.56 % - 06.95 %]	No effect on splicing	0	0	0	0	0,05	0
NM_012433.3:c.2206C>T	p.R736C	0	0	08.25 % [04.79 % - 13.05 %]	No effect on splicing	0	0	0	0	0,21	0
NM_012433.3:c.1759T>A	p.Y587N	0	0	26.62 % [19.83 % - 34.34 %]	Alteration of an exonic splicing regulatory element	0	0	0	0	0	0
NM_012433.3:c.1190G>A	p.R397H	0	0	30.67 % [23.41 % - 38.71 %]	Alteration of an exonic splicing regulatory element	0	0	0	0	0	0,01
NM_012433.4:c.1108C>T	p.P370S	0	0	05.05 % [02.45 % - 09.09 %]	No effect on splicing	0	0	0	0	0	0
NM_012433.4:c.1109C>T	p.P370L	0	0	05.05 % [02.45 % - 09.09 %]	No effect on splicing	0	0	0	0	0	0

Supplementary Table 2. ***In silico*** **prediction of the pathogenicity of** ***SF3B1*** **missense variants** using the following algorithms: SIFT, PolyPhen2, Mutation Taster, MetaSVM, CADD, REVEL, Alphamissense. D = Deleterious, T = Tolerated.

Individual	Variant c. (NM_012433.3)	Variant p.	SIFT	PolyPhen2	Mutation Taster	MetaSVM	CADD	REVEL	Alphamissense
1	c.2486A>G	p.N829S	T	possibly_damaging	D	T	24	0.45	0.7316
3	c.3085G>A	p.E1029K	D	probably_damaging	D	D	29.2	0.56	0.9987
4	c.2716G>A	p.E906K	D	possibly_damaging	D	D	32	0.54	0.9926
5	c.1606C>A	p.L536I	D	probably_damaging	D	T	26.7	0.46	0.9827
6	c.3686C>G	p.A1229G	T	benign	D	T	23	0.43	0.3611
7	c.2164G>A	p.E722K	D	probably_damaging	D	D	28.5	0.48	0.9987
8, 16	c.2815C>T	p.R939C	D	probably_damaging	D	D	28.6	0.45	0.9952
11	c.2339C>T	p.P780L	D	probably_damaging	D	D	27.9	0.58	0.9932
14	c.2107A>C	p.T703P	D	probably_damaging	D	D	27.1	0.73	0.9953
17	c.2221A>C	p.K741Q	D	probably_damaging	D	D	31	0.58	0.9984
18	c.1837A>G	p.M613V	D	possibly_damaging	D	T	25	0.52	0.964
19	c.2206C>T	p.R736C	D	benign	D	T	25.6	0.38	0.935
20	c.1759T>A	p.Y587N	D	probably_damaging	D	D	28.8	0.84	0.975
24	c.1190G>A	p.R397H	D	probably_damaging	D	D	27.8	0.54	0.61
25	c.1108C>T	p.P370S	D	probably_damaging	D	D	29.1	0.699	0.978
26	c.1109C>T	p.P370L	D	probably_damaging	D	D	31	0.798	0.959

Supplementary Table 3. ACMG classification of the *SF3B1* variants. Variants were classified according to the ACMG guidelines[2]. For the variants with sufficient functional data, classification was updated accordingly. dn: de novo.

Variant c.	Variant p.	Inheritance	Criteria	Classification (without functionnal data - PS3)	Classification (with functionnal data - PS3)
c.2486A>G	p.N829S	dn	PS3, PM2, PM6, PP2	VUS	Likely Pathogenic
c.3085G>A	p.E1029K	dn	PM2, PM6, PP2, PP3	Likely Pathogenic	Likely Pathogenic
c.2716G>A	p.E906K	dn	PM2, PM6, PP2, PP3	Likely Pathogenic	Likely Pathogenic
c.1606C>A	p.L536I	dn	PM2, PM6, PP2, PP3	Likely Pathogenic	Likely Pathogenic
c.3686C>G	p.A1229G	dn	PM2, PM6, PP2	VUS	VUS
c.2164 G>A	p.E722K	dn	PS3, PM2, PM6, PP2, PP3	Likely Pathogenic	Pathogenic
c.2815C>T	p.R939C	dn	PM2, PM6, PP2, PP3	Likely Pathogenic	Likely Pathogenic
c.2587C>T	p.Q863*	dn	PVS1-S, PM2, PM6	Likely Pathogenic	Likely Pathogenic
c.2938G>T	p.E980*	dn	PVS1-S, PM2, PM6	Likely Pathogenic	Likely Pathogenic
c.416-1G>T	p.?	dn	PVS1-S, PM2, PM6	Likely Pathogenic	Likely Pathogenic
c.2339C>T	p.P780L	dn	PS3, PM2, PM6, PP2, PP3	Likely Pathogenic	Pathogenic
c.1428_1429insTA	p.K477*	dn	PVS1-S, PM2, PM6	Likely Pathogenic	Likely Pathogenic
c.469C>T	p.R157*	dn	PVS1-S, PM2, PM6	Likely Pathogenic	Likely Pathogenic
c.2107A>C	p.T703P	dn	PM2, PM6, PP2, PP3	Likely Pathogenic	Likely Pathogenic
c.456C>A	p.Y152*	dn	PVS1-S, PM2, PM6	Likely Pathogenic	Likely Pathogenic
c.2221A>C	p.K741Q	dn	PM2, PM6, PP2, PP3	Likely Pathogenic	Likely Pathogenic
c.1837A>G	p.M613V	dn	PM2, PM6, PP2, PP3	Likely Pathogenic	Likely Pathogenic
c.2206C>T	p.R736C	dn	PM2, PM6, PP2	VUS	VUS
c.1759T>A	p.Y587N	dn	PM2, PM6, PP2, PP3	Likely Pathogenic	Likely Pathogenic
c.2427_2428del	p.E809Dfs*7	maternally inherited	PVS1-S, PM2	Likely Pathogenic	Likely Pathogenic
c.2080del	p.V695Wfs*34	paternally inherited	PVS1-S, PM2	Likely Pathogenic	Likely Pathogenic
c.496C>T	p.R166*	paternally inherited	PVS1-S, PM2	Likely Pathogenic	Likely Pathogenic
c.1190G>A	p.R397H	unknown	PM2, PP2, PP3	VUS	VUS
c.1108C>T	p.P370S	dn	PM2, PM6, PP2, PP3	Likely Pathogenic	Likely Pathogenic
c.1109C>T	p.P370L	dn	PM2, PM6, PP2, PP3	Likely Pathogenic	Likely Pathogenic

Supplementary Table 4. List and occurrence of the NDD-associated missense *SF3B1* variants that have been reported in the COSMIC database (2024).

Variant	Sample Name	Transcript	Primary Tissue	Tissue Subtype 1	Histology	Somatic Status
T703P	P-0008766-T01-IM5	ENST00000335508.10	biliary_tract	bile_duct	carcinoma	Confirmed Somatic
E722K	HCC2998	ENST00000335508.10	large_intestine	colon	carcinoma	Confirmed Somatic
E722K	HCC2998	ENST00000335508.10	large_intestine	NS	carcinoma	Previously Reported
E722K	TCGA-CA-6717-01	ENST00000335508.10	large_intestine	colon	carcinoma	Confirmed Somatic
R736C	TCGA-D1-A17A-01	ENST00000335508.10	endometrium	NS	carcinoma	Confirmed Somatic
R736C	2938084	ENST00000335508.10	eye	uveal_tract	malignant_melanoma	Previously Reported
R736C	5-143	ENST00000335508.10	small_intestine	duodenum	carcinoma	Confirmed Somatic
R736C	61	ENST00000335508.10	small_intestine	duodenum	adenoma	Confirmed Somatic
K741Q	TCGA-MI-A75I-01	ENST00000335508.10	liver	NS	carcinoma	Confirmed Somatic
E906K	P-0008684-T01-IM5	ENST00000335508.10	biliary_tract	bile_duct	carcinoma	Confirmed Somatic
E906K	TCGA-CN-6011-01	ENST00000335508.10	upper_aerodigestive_tract	head_neck	carcinoma	Confirmed Somatic
R939C	BL-16-A36234	ENST00000335508.10	large_intestine	NS	carcinoma	Unknown
E1029K	CHG-2013-27553T	ENST00000335508.10	liver	NS	other	Confirmed Somatic
E1029K	TCGA-DK-A2I4-01	ENST00000335508.10	urinary_tract	bladder	carcinoma	Confirmed Somatic
R397C	P-0001115-T01-IM3	ENST00000335508.10	endometrium	NS	carcinoma	Confirmed Somatic
R397C	TCGA-B5-A11E-01	ENST00000335508.10	endometrium	NS	carcinoma	Confirmed Somatic
R397C	TCGA-G4-6586-01	ENST00000335508.10	large_intestine	colon	carcinoma	Confirmed Somatic
P370L	P-0004683-T01-IM5	ENST00000335508.10	central_nervous_system	brain	glioma	Confirmed Somatic
P370L	TCGA-EE-A3AE-06	ENST00000335508.10	skin	NS	malignant_melanoma	Confirmed Somatic

Supplemental Table 5. Enrichment in RNA binding protein (RBP) sites at alternatively spliced junctions (A3'SS and SE) using the rMPAS2 (RNA Map Analysis and Plotting Server 2) software. Only enriched motifs (up and down) common to E722K, P780L and N829S variants are shown in the table.

Analysed region	Enrichment direction compared to background	Name of RBP protein	Corresponding motif
A3'SS region	up	SRSF1	GGA[GC]G[AG][ACG]
	down	SRSF9	[GT]G[AG][AT]G[GC][AC]
		RBM47	GATGA[AT]
		SRp40	[CT][AG]C[AG][GT][AC]
		SRp20	CTC[GT]TC[CT]
		PCBP2	CC[CT][CT]CC[ACT]
		SRSF1	AGGA[GC][AC]
SE region (exon 5')	up	Hup	TT[AT]GTTT
		HNRNPA1	[AGT]TAGGG[AT]
		PTBP1	[ACT][CT]TTT[CT]T
		RBM42	AACTA[AC]G
		PCBP1	CC[AT][AT][ACT]CC
	down	RBMS3	[AC]TATA[GT][AC]
		CNOT4	GACAGA
SE region (exon 3')	up	SRSF1	G[AG]AGGA
		PABPC3	[AG]AAAAC[AC]
		A1CF	[AT]TAATT[AG]
	down	HNRNPA1L2	[AGT]TAGGG[AT]
		RBMS3	[AC]TATA[GT][AC]

Supplementary references

1. Baux, D. *et al.* MobiDetails: online DNA variants interpretation. *Eur. J. Hum. Genet. EJHG* 29, 356–360 (2021).
2. Rentzsch, P., Witten, D., Cooper, G. M., Shendure, J. & Kircher, M. CADD: predicting the deleteriousness of variants throughout the human genome. *Nucleic Acids Res.* 47, D886–D894 (2019).
