# Supplementary material for: De novo variants in the splicing factor gene SF3B1 are associated with neurodevelopmental disorders
Source: Nat Commun. 2026 Jan 23;17:1569. doi: 10.1038/s41467-026-68284-9 (PMC12902031; doi:10.1038/s41467-026-68284-9)
Supplement: Supplementary file 2 — Description of Additional Supplementary Files [file 41467_2026_68284_MOESM2_ESM.pdf]

Description of Additional Supplementary Files

File Name: Supplementary Data 1

Description: Extended clinical table.

File Name: Supplementary Data 2

Description: List of differentially expressed genes identified using the R package DESeq2. Differential expression was assessed with the Wald test, and p-values were adjusted for multiple comparisons using the Benjamini– Hochberg (BH) method. Genes with an adjusted p-value < 0.05 were considered significantly differentially expressed.

File Name: Supplementary Data 3

Description: Pathway enrichment analysis was performed on differentially expressed transcripts (DETs) using GO, KEGG, Reactome, and WikiPathways terms for variants P780L, N829S, and E722K. Enrichment was assessed with gprofiler2, which applies a modified Fisher's exact test to evaluate overrepresentation of pathway terms. Results were corrected for multiple testing using the false discovery rate (FDR).

File Name: Supplementary Data 4

Description: List of oligonucleotides used in this study
